# The utility of surveillance peritoneal dialysis effluent cultures following completion of PD peritonitis treatment: a quality improvement report

**DOI:** 10.1093/ckj/sfad307

**Published:** 2024-01-31

**Authors:** Ana Sanchez-Escuredo, Mina Kashani, Jeffrey Perl

**Affiliations:** Division of Nephrology, St. Michael's Hospital, Unity Health, University of Toronto, ON, Canada; Division of Nephrology. Hospital Moises Broggi, Barcelona, Spain; Division of Nephrology, St. Michael's Hospital, Unity Health, University of Toronto, ON, Canada; Division of Nephrology, St. Michael's Hospital, Unity Health, University of Toronto, ON, Canada

To the Editor,

Peritoneal dialysis (PD) associated peritonitis is a leading cause of premature transition to hemodialysis. Successful treatment with antibiotics alone occurs in 85% of episodes [1]. Relapsing (peritonitis with the identical organism within 4 weeks of the initial episode) and repeat (peritonitis with an identical organism after 4 weeks of the initial episode) peritonitis episodes remain an ongoing concern despite completion of antibiotic treatment. One postulated mechanism for relapsing/repeating episodes is the development of a PD catheter bacterial biofilm impenetrable to antimicrobials, often necessitating PD catheter removal.

Current peritonitis surveillance practices recommends a follow-up PD effluent leukocyte count and culture 2–3 days after the initiation of appropriate antibiotic therapy to ensure a declining leukocyte count and sterilization of the PD effluent [2]. Repeating the PD effluent leukocyte count and culture is only suggested if no clinical response occurs after 3–5 days of treatment to identify refractory episodes potentially requiring PD catheter removal [3].

Whether surveillance of the PD effluent culture following peritonitis treatment can identify persistent growth of organisms prior to the development of a relapsing peritonitis episode is not known. Historically, our center has performed PD effluent cell count and culture 1 week after treatment completion to confirm the resolution of the peritonitis episode for those patients with a successfully treated PD associated peritonitis, even though it is not stated in the ISPD guidelines, to avoid potential risk of a further relapsing event which may require PD catheter removal. Here, we audited how often our current practice resulted in identification of effluent culture positivity.

We reviewed all peritonitis episodes at a single center between June 2010 and December 2022. Peritonitis definition and treatment was in accordance with ISPD criteria and antimicrobial agents and duration, microorganism-specific [2]. PD effluent cell count and effluent cultures were repeated 48 h after the diagnosis, at day 5, and 1 week after antibiotic completion. All patients received prophylactic antifungal therapy with nystatin during antibiotic treatment and up to one week following antibiotic completion. This initiative was formally reviewed by institutional authorities at Unity Health Toronto and deemed to neither require Research Ethics Board approval nor written informed consent from participants.

**Figure 1:**
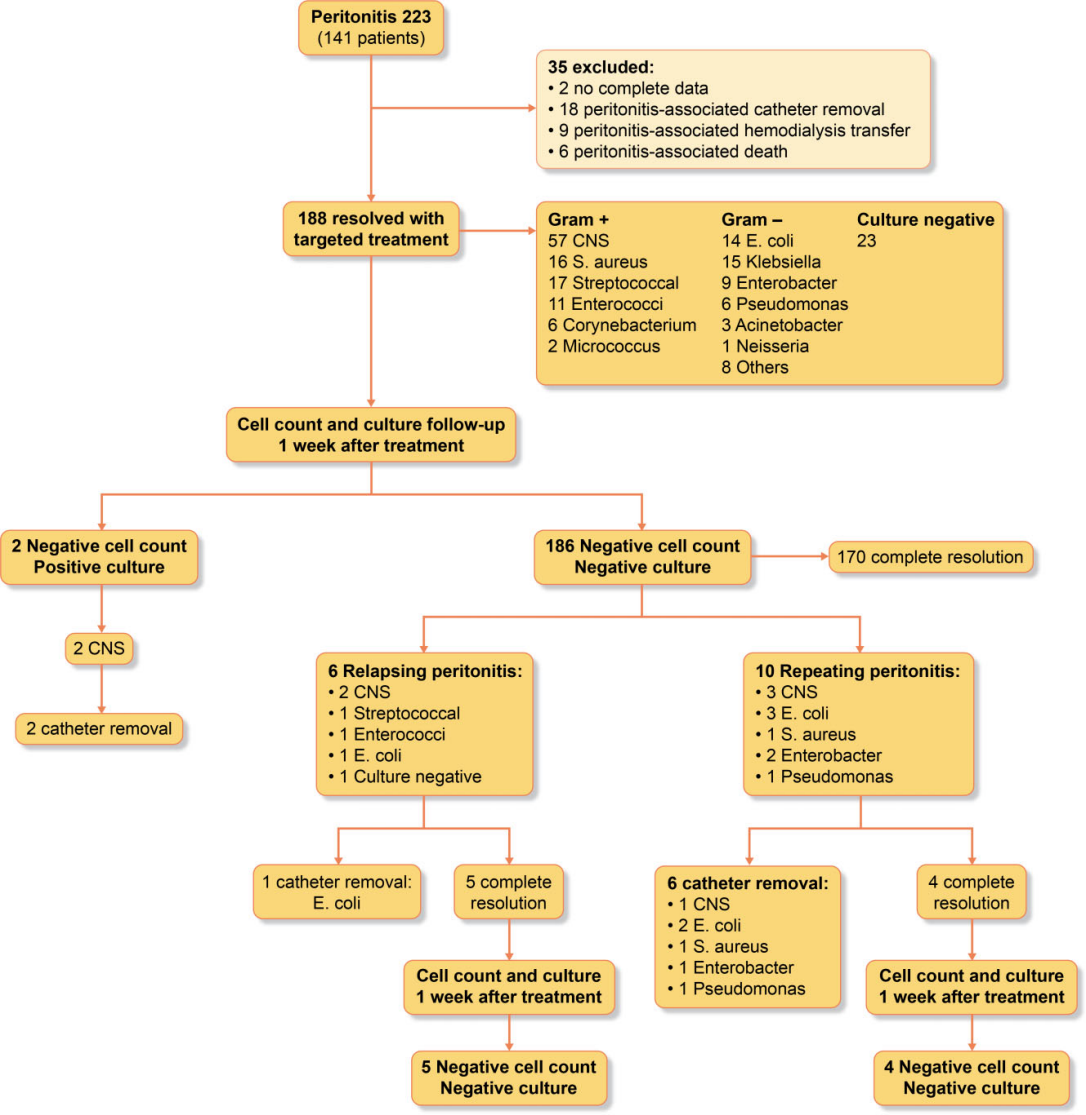
Outcomes of surveillance PD cell count and effluent culture results after one week of completion of antimicrobial therapy. Peritonitis-associated catheter removal: removal of PD catheter as part of the treatment. Peritonitis-associated hemodialysis transfer: transfer from PD to hemodialysis for any period as part of the treatment. Peritonitis-associated death: Death occurring within 30 days of peritonitis onset or death during hospitalization due to peritonitis.

In total, 223 peritonitis episodes (141 patients) were reported. Thirty-five peritonitis episodes (16%) were excluded due to complications arising during treatment or missing data (Fig. 1). Of the 188 resolved peritonitis episodes, 57% were Gram positive, 30% Gram negative and 12% culture negative peritonitis. Among patients completing medical treatment for peritonitis, 6 (3%) presented with relapsing peritonitis and 10 (6%) with repeating peritonitis.

Only 2/188 patients (1%) had positive PD effluent surveillance cultures; both for coagulase negative staphylococcus (CNS), the same organism as the initial episode. In one case, the patient presented for surveillance cultures after treatment with a positive CNS culture without abdominal pain and a negative cell count. The next day he presented with abdominal symptoms, had a positive culture with an elevated neutrophil-predominant cell count and antibiotic treatment was initiated. We did not find out the asymptomatic surveillance culture was positive until after the patient presented the following day with symptoms. In the second instance, the patient presented with a CNS positive culture with negative cell count and no clinical symptoms. One was considered a relapsing episode and the second case, potential prevention of a relapsing peritonitis. For both, 2 weeks of antibiotics alone failed to sterilize the PD effluent, ultimately resulting in PD catheter removal. No surveillance cultures were positive among the patients in the relapsing and repeating group after treatment completion.

In our quality improvement audit, we found that the yield of surveillance cultures and cell count following a PD-associated peritonitis episode following treatment is extremely low at 1% of all successfully treated episodes and does not support the continuation of our current practice. In both cases, the positive surveillance culture was due to CNS (3.5% of all CNS episodes). CNS is the most common cause of peritonitis and device-related infections. Despite CNS peritonitis having excellent treatment cure rates with antibiotics alone, reported risks of relapsing and repeat events are around 12% often after antibiotic treatment cessation [4, 5]. Bacteria-derived DNA fragment levels in PD effluent after the completion of antibiotics are persistently elevated amongst patients developing relapsing peritonitis [6]. Unique to CNS is its ability to produce biofilms adhering to device surfaces, in particular, with the presence of biofilm-related genes *mecA* and *icaAD*, which encodes a low affinity penicillin-binding protein not affecting CNS deep in the biofilm, resulting in a nidus for relapse [5].

Our present results do not support a high yield of repeating a post 1-week surveillance cell count or culture following a successfully treated PD-associated peritonitis episode. We found that a positive culture after peritonitis treatment conclusion rarely occurred but only for 2/59 (3.5%) of all CNS peritonitis episodes in our cohort. Given the single-center nature of our study, further confirmation may be needed with ongoing multi-center data collection (among centers with varying peritonitis, relapse and repeat event rates particular higher CNS peritonitis rates) to explore the utility of repeat peritoneal effluent culture sampling after peritonitis treatment, particularly for patients with initially presenting CNS peritonitis. Conducting surveillance at varying times after treatment completion beyond just one week, such as one month as well, may also be entertained. However, given our findings, we will discontinue this practice at our center.
